# Possibilities of alternative generation II biotests at *Artemia*

**DOI:** 10.2478/v10102-009-0008-0

**Published:** 2009-06

**Authors:** Petr Dvořák, Michal Žďárský, Katarína Beňová

**Affiliations:** 1 Department of Biochemistry, Chemistry and Biophysics, Faculty of Veterinary Hygiene and Ecology, University of Veterinary and Pharmaceutical Sciences, Brno, Czech Republic; 2 Department of Biology and Genetics, University of Veterinary Medicine, Košice, Slovak Republic

**Keywords:** *Artemia salina*, *Artemia franciscana*, microbiotests, animal protection

## Abstract

The meaning of alternative biotests is described and discussed. The paper also deals with the possible application of the developmental studies of the sea *Artemia franciscana* nauplinus. Five-day biotests including the validation criteria are described. The possibilities of the biotests are very wide. Additionally to the standard applications in ecotoxicology, there is a possibility of modelling pharmacological experiments or monitoring the effects of ionizing radiation and the interaction with other chemicals.

## Introduction

In biology the phenomena are so complicated and the mutual interactions so frequent that the theoretical principles are only temporary and hypothetic. Even very precise human deductions are uncertain and they require the experimental verification. Two apparently incompatible tendencies are known from the middle of the last century in biomedical research. On one hand, this is an attempt to understand etiopathogenesis of many diseases and successively the development of enormous quantity of new substances with the need of their testing, and on the other hand there is pressure of the society which calls at least for mitigation of test animal suffering (Russell and Burch, 1959). This demand has led to the creation of the 3 R concept (i.e. Reduction, Refinement and Replacement). This strategy also involves substituting the test animals by cell or tissue cultures and lower organisms, for example, invertebrates or microorganisms (defined according to the Council of Europe 1976). The important difference which distinguishes the biological tests from other tests is in the complexity of live systems that cannot be simplified. If we use tissue or cells in a research, it is only a study of partial systems not the study of a simplified system (Pazourek, 1992).

The environment including water ecosystems is very often contaminated by low concentrations of various chemical compounds of foreign origin (Beňová *et al*., 2007). Although the resistance of test organisms to various substances is relatively well known, it is impossible to determine toxicity of mixtures of various components. Similar situation is also in medicine where information about the long-term effects of combined pharmaceuticals or the effects of their residuals on water organisms is relatively very poor (Sklenář *et al*., 2006). The foreign substances in low concentrations can interact with some physical factors. The effects can occur in some time, and their subacute or chronic effects can be expected (Dvořák and Beňová, 2002; Beňová *et al*., 2006).

## Biotests

The tests to monitor the interactions should meet the following requirements: sufficient sensitivity to foreign substances; subacute to long-term nature in the time; high homogeneity of the individuals who will be relatively less sensitive to the external conditions of the experiment; high reproducibility of the experiments; as high as possible closeness of the system; simplicity of production and verification; price availability; and simple evaluation and simple statistical processing.

The standard biotests performed on fish or invertebrates have some disadvantages. From the methodological point of view, the biotests are highly demanding for both the time and the personnel qualification. Hence such experiments are too expensive. The generation II tests solve the problem mentioned. These are the alternative microbiotests in which unicellular or small multicellular organisms are exposed to liquid nitrogen when the specific effect is measured. Various microorganisms, e.g. bacteria, mushrooms, algae, protozoa and invertebrates, are used (Blaise, 1991). The principle of so-called “toxkits” can lead to incubation of the test animals from the resting eggs (marked as cysts) within 24 hours before starting the test. This method can avoid the time-consuming and spatially demanded cultivation of the live organisms. ROTOXKIT F and M, THAMNOTOXKIT F, ARTOXKIT M and others are manufactured in large series. However, the greater production represents the research which is financially demanded. The affordable tests on *Artemia franciscana* (previously *A. salina*) discussed in this contribution, have been performed since 1992, that is, they started before Czech Republic approached to the import of the toxkit.

## Biotests using brine shrimps of genus Artemia

One invertebrate routinely used in the different biotests for a long time is genus *Artemia* (order Anostraca order, class Branchiopoda) that forms an independent group of nauplii stages. The population of *Artemia* can be found in about 500 salt lakes and salt works in the temperate, sub-tropical, and tropical climate zones. Because they are adapted to high salinity, their biotops are characterized by the minimum diversity of genera and the absence of predators and food competitors, leading to the mono-culture of *Artemia*. *Artemia* are able to to have a good run at the salinity of 70 g.L^–1^ and survive even at the concentrations of 250 g.L^–1^ (Ruppert, 2003). *Artemia* are provided with the unique ability to survive even in the environment with a high salinity and to withstand the osmotic pressure. The nauplii stages receive water and excrete sodium and chloride ions by means of the cervical organs. The transport of sodium is carried out by Na-K-ATPase via the secretion cells (Conte *et al*, 1972). More than 600 papers have been published and registered in the “BioMedNet” database that reflect the significance of *Artemia franciscana*, Kellogg (1906), (formely *A. salina*) in biology and medicine.

“Salt water” (ASW) which consists of the chemicals with p.a. purity is used for the long-term experiments. All chemicals used for the preparation of ASW were of Analytical Grade (Merck, FRG). (g.L^–1^): 23.9 NaCl; 10.83 MgCl_2_ · 6H_2_O; 2.25 CaCl_2_ · 6H_2_O; 0.68 KCl; 9.06 Na_2_SO_4_ · 10 H_2_O; 0.2 NaHCO_3_; 0.04 SrCl_2_ · 6H_2_O; 0.099 KBr; 0.027 H_3_BO_3_ (Dvořák 1995). This composition represents a salinity of 49 g.L^–1^, high purification ability, at pH-value of 7.6 ± 0.1 (Dvořák, 1999). Irsa (1995) stated that ASW is stable for two months if kept in cool and dark room. For the application in pharmacotoxicology, it is better to use much lower salinity, e.g. 9 g.l^–1^ (Sklenář *et al*., 2006). This is very important from the point of view of better solubility of the tested substances due to a lower ion salt density. For the solids insoluble in water, low toxicity diluents can be applied. For example, dimethylsulfoxide with a concentration of 12 g.L^–1^ was used in our experiments.

Science of nowadays requires the mathematical planning of the experiments, that means a selection of the optimized experimental scheme that can provide us with the utmost information while the minimum number of the tests is performed. The planning of experiments can allow: (1) Reducing the experimental error and eliminating the effects of random factors. (2) Reducing the number of tests and getting the valid answer with the specified accuracy to the pre-selected question. (3) Accepting the solution based on the precisely specified rules. The experimental methodology is given by: (1) Repetition of the experiments. (2) Creation of the blocks under the same conditions. (3) Data validation at the beginning and at the end of experiments. (4) Use of the random procedures to eliminate the random error effect.

The biological subjects are extremely complicated, however, the system can be defined relatively simply. The experimental formation of an insulated system (no exchange of energy, mass and information) may hardly be used in practice, and hence the closed systems are used (no exchange of mass with the environment). To perform experiments in the open systems is very difficult. There exist an indefinite quantity of random effects hardly to evaluate.

Measure of variability decides about the scope of experimental and control sets. This is the accompanying symptom of all biological experiments while we have to distinguish between the interindividual and intraindividual variability. The experimenter tries to reduce the measure of variability as much as possible because the variability makes difficult to get the unique results as well as the reproducibility of the experiments. The next variability impact is an uncontrolled effect of the environment. The measure of variability in a form of experimental errors can be mathematically affected (Dvořák, 1999).

The subacute biotest at *Artemia franciscana* is suitable for the requirements above mentioned. According to the nature of incubation of nauplii stages from cysts, this test can be ranked among the generation II tests. *Artemia franciscana*, known as the “brine shrimps”, are suitable for the monitoring of toxicity because they are very sensitive to many chemical substances, and moreover, morphological changes can be monitored. Extreme durability of the resting eggs (up to some years), high hatching performance (90% and more within 24 hours at temperature of 25 °C) and last but not least even availability and price are advantageous. Depending on the quality, one gram contains from 220,000 to 260,000 resting eggs. We can purchase for 50 EUR approximately 250 million individuals of a homogenous population as resting eggs (as called as cysts). The best stages come from Salt Lake (Utah, USA) where they are sorted and packed into evacuated tins for aquaristic purposes.

Fifty individuals are used in each test for each concentration of tested substances. The Petri dishes are used. Ten specimens in total volume of 10 ml ASW including the tested substances are put into each Petri dishes. The number of live individuals is counted every 24 hours. The killed individuals need not be removed because due to their negligible weight they will not affect the next experiment run (Dvořák, 1995). To standardize the experiment, the nauplii stages were maintained in stable environment and without feeding. Temperature is the only factor that causes some troubles. The temperature fluctuation should be lower than 1 °C. Otherwise the test duration can be reduced or extended. The test reproducibility always relates to this fact. There is one solution – air-conditioned boxes. The lethality must be evaluated separately after completing each test and compared with the control group. The range of individual attempts is limited due to the time demands during readout. The time span between the start and end of readout should not be too long. The results read by more than one person can lead to an undesired variability.

Validation for the subacute test should be secured by the mortality of the control group lower than 10%. However, this criterion is designed for the daphnia tests with an exposure of up to 48 hours without any feeding, or for the tests when the monitored objects are optimally fed. Because *Artemia* are hungry, they exhaust their energy reserves and the strict criterion within 120 hours cannot be always met. On the other hand, *Artemia* are very sensitive within this period for the presence of toxic agens and the maximum differences between the mortality of the control and tested groups are achieved.

To monitor a long-term effect of the substances on the organisms it is necessary to extend as most as possible the viability of tested organisms under the standard conditions. Because *Artemia* start to perish after 96 hours for food shortage, it is necessary to supply the standard energy sources. This can provide the addition of 3% glucose that prolongs the test up to ten days (Dvořák *et al*., 2005). In this test of prolonged toxicity the more moderate validation criteria were designed for the exposure longer than 120 hours, i.e. 20% mortality in the control groups because glucose does not represent full nutritive value. The test should be completed as soon as mortability would exceed 20%.

For the monitoring of unstable substances the validation criterion is to determine the concentration of substances in the solution at the beginning and at the end of the experiment.

The widest use of the above mentioned test is in ecotoxicology. Because it is possible to monitor high numbers of specimens at the same time (up to 1,000), we can study the mutual interactions of chemical substances and their physical factors (Dvořák, 1999; Dvořák and Beňová, 2002; Beňová *et al*., 2006; Beňová *et al*., 2007).

## The test in practice

The example of the generation II biotest application at *Artemia franciscana* in pharmacology represents the utilization in the primary toxicity screening of the new synthesized purine inhibitors of cyclin-dependent kinases. Toxicity was compared with toxicity of olomoucin and also with toxicity of risk elements – chromium, cadmium, zinc and boron. The experiment was designed as the toxicity test in the environment with 0.9% salinity (Sklenář *et al*., 2006).

The cosmic radiation effect on *Artemia franciscana* cysts was studied during the unique Biostack project performed on Apollo 16 board. Hatching occurred only at 10 percent at cysts exposed to cosmic radiation (Ruther 1974).

Study on lethality of *Artemia franciscana* depending on the dose of gamma ionizing radiation determined LD_50_ of 96 hours when exposed to 600–700 Gy (Dvořák and Beňová, 2002) which even corresponded to phylogenetic genus classification. The study which monitored morphologic changes at *Artemia franciscana* after gamma radiation exposure was presented at the 2^nd^ Radiobiological Conference at Košice. Intestinal epithel is the most sensitive, and the changes of epitelial cells were already observed with the nauplii exposed to a dose of 100 Gy. In individuals exposed to a dose of 1,000 Gy intestinal epithel was completely destroyed. Loss of segmentation in thoracal area and cease of appendiceal formation were the next significant morphological changes (Dvořák *et al*., 2004).

The alternative biotests cannot replace the conventional tests in a full range at experimental mammals, but they can reduce remarkably their numbers.
